# DNA extraction protocols cause differences in 16S rRNA amplicon sequencing efficiency but not in community profile composition or structure

**DOI:** 10.1002/mbo3.216

**Published:** 2014-09-26

**Authors:** Benjamin E R Rubin, Jon G Sanders, Jarrad Hampton-Marcell, Sarah M Owens, Jack A Gilbert, Corrie S Moreau

**Affiliations:** 1 Committee on Evolutionary Biology, University of ChicagoChicago, Illinois; 2 Department of Science and Education, Field Museum of Natural HistoryChicago, Illinois; 3 Department of Organismic and Evolutionary Biology, Harvard UniversityCambridge, Massachusetts; 4 Institute of Genomic and Systems Biology, Argonne National LaboratoryLemont, Illinois; 5 Department of Ecology and Evolution, University of ChicagoChicago, Illinois; 6 Computation Institute, University of ChicagoChicago, Illinois

**Keywords:** 16S rRNA, ants, DNA extraction, Earth Microbiome Project, host-associated bacteria, insects, microbiome

## Abstract

The recent development of methods applying next-generation sequencing to microbial community characterization has led to the proliferation of these studies in a wide variety of sample types. Yet, variation in the physical properties of environmental samples demands that optimal DNA extraction techniques be explored for each new environment. The microbiota associated with many species of insects offer an extraction challenge as they are frequently surrounded by an armored exoskeleton, inhibiting disruption of the tissues within. In this study, we examine the efficacy of several commonly used protocols for extracting bacterial DNA from ants. While bacterial community composition recovered using Illumina 16S rRNA amplicon sequencing was not detectably biased by any method, the quantity of bacterial DNA varied drastically, reducing the number of samples that could be amplified and sequenced. These results indicate that the concentration necessary for dependable sequencing is around 10,000 copies of target DNA per microliter. Exoskeletal pulverization and tissue digestion increased the reliability of extractions, suggesting that these steps should be included in any study of insect-associated microorganisms that relies on obtaining microbial DNA from intact body segments. Although laboratory and analysis techniques should be standardized across diverse sample types as much as possible, minimal modifications such as these will increase the number of environments in which bacterial communities can be successfully studied.

## Introduction

The reduction in sequencing-associated costs required for analyzing microbial community structure using the 16S rRNA gene has caused a rapid increase in the number and breadth of these studies (Knight et al. 2012). Some of the best-known applications of these techniques to host-associated communities have focused on the human microbiome (Costello et al. 2009; Caporaso et al. 2011; Wu et al. 2011; Schloissnig et al. 2013), but as characterizing novel bacterial communities has become cheaper and easier (Liu et al. 2007; Andersson et al. 2008; Bartram et al. 2011; Caporaso et al. 2012), studies have begun to explore the microbiota of such diverse environments as mammal and honeybee guts (Muegge et al. 2011; Martinson et al. 2012), leaf-cutter ant fungal gardens (Suen et al. 2010; Aylward et al. 2012), marine systems (Gilbert et al. 2012; Gibbons et al. 2013), and even oil plumes (Hazen et al. 2010). The increase in breadth of these investigations has shifted attention toward optimization of sample processing to increase the number of samples that can be examined and directly compared; sequencing is no longer the bottleneck it once was. Sharing of standard operating procedures for sample preparation and data analysis has been an invaluable part of these efforts, making this area of study available to a broad array of researchers (e.g., Caporaso et al. 2012; Engel et al. 2013; Kozich et al. 2013; Schloss et al. 2011; http://www.earthmicrobiome.org/emp-standard-protocols/; http://www.mothur.org/wiki/Analysis_examples; http://qiime.org/tutorials/index.html). Optimization of DNA extraction methods is of particular interest when developing protocols as this is among the first steps in the analysis of microbial diversity, and therefore can have a significant influence on the structure and diversity of the recovered community profile (de Lipthay et al. 2004; Carrigg et al. 2007; Feinstein et al. 2009; Willner et al. 2012). Indeed, certain protocols can even systematically introduce contaminants (Willner et al. 2012).

To help avoid problems of extraction bias, recent initiatives to investigate microbiomes on a large scale, including the Earth Microbiome Project (EMP) (Gilbert et al. 2010a, 2010b, 2011) and the Human Microbiome Project (Turnbaugh et al. 2007; The NIH HMP Working Group 2009; Knight et al. 2012), have placed a premium on standardization of sample handling and processing techniques. This standardization removes biases associated with different extraction protocols, PCR reactions, and sequencing platforms; however, the exclusion of those samples not compatible with the chosen standards limits the communities that can be examined.

Insect-associated bacterial communities have recently proven to be fruitful subjects of study (e.g., Martinson et al. 2012; Jones et al. 2013; Kautz et al. 2013a, 2013b; Hu et al. 2014; Jing et al. 2014; Sanders et al. 2014). Despite interest in making broad comparisons across many taxa, standard methods for DNA extraction have not been established in this group (Russell et al. 2009; Colman et al. 2012; Jones et al. 2013). Rigid exoskeletons offer an additional challenge to DNA extraction often not considered when designing general protocols. While many microbiome studies examine animal feces, similar materials are often difficult to obtain from insects. Thus, dissected gut tissues, abdominal segments containing the entire digestive tract, or whole insects have been used instead (Jones et al. 2013; Kautz et al. 2013b). Here, we use several ant species as focal organisms to compare the quantity of bacterial DNA obtained from standard methods of nucleic acid extraction. We then examine how each technique affects the microbial community structure and composition using 16S rRNA amplicon sequencing of the V4 region following the EMP standard protocols (http://www.earthmicrobiome.org/emp-standard-protocols/16s/). Ants are an ideal group in which to study these issues as closely related and communally living individuals are easy to collect in large numbers and many species possess armored exoskeletons. We propose that the methodological comparison provided here is an excellent proxy for insects in general.

## Experimental Procedures

### DNA extraction

We compare four DNA extraction protocols in this study: (1) Phenol–chloroform (Sigma-Aldrich Co. LLC, Saint Louis, MO), (2) the Qiagen DNeasy Blood & Tissue Kit (Qiagen Inc., Valencia, CA), (3) the PowerSoil DNA Isolation Kit (MO BIO Laboratories, Carlsbad, CA), and (4) the PowerSoil DNA Isolation Kit with the addition of a tissue homogenization and digestion step (modified PowerSoil).

Following standard protocols, the phenol–chloroform extractions included a tissue homogenization step using a Qiagen TissueLyser. Tissues were pulverized dry with Qiagen tungsten carbide beads for 20 sec at 30 beats per second. Subsequently, 250 *μ*L of buffer A (200 mmol/L Tris-HCl pH 8.8, 60 mmol/L NaCl, 10 mmol/L EDTA [ethylenediaminetetraacetic acid], 0.15 mmol/L spermine, and 0.15 mmol/L spermidine) were added to the homogenized ants and nucleases were inactivated by a 15 min incubation at 65°C. Then, 250 *μ*L buffer B (200 mmol/L Tris-HCl pH 8.8, 30 mmol/L EDTA, and 2% SDS) were added and samples were incubated for 10 min at 65°C. To fully digest ant tissues, 100 *μ*g of proteinase K were added, and the samples were again incubated at 56°C for at least 1 h. Two phenol–chloroform (phenol/chloroform/isoamyl alcohol, 25:24:1, pH 8.0) washes and a third chloroform wash to remove residual phenol, were performed. DNA was precipitated with 50 *μ*L of 5 mol/L NaCl and 1 mL of ice-cold 100% ethanol. Pellets were washed with 70% ethanol and resuspended in 50 *μ*L of Tris-EDTA (TE).

The Qiagen extractions included an identical tissue homogenization step to the phenol–chloroform approach, as suggested in the manufacturer's protocol. All subsequent steps were completed using the standard manufacturer's protocols, including overnight proteinase K digestion. Elutions were done with 50 *μ*L buffer AE.

The standard PowerSoil extraction was performed according to the manufacturer's protocol with the addition of a 20 min incubation at 65°C after addition of solution C1, as suggested by the EMP (http://www.earthmicrobiome.org/emp-standard-protocols/16s/). Samples were eluted in 50 *μ*L of solution C6. Explicit tissue homogenization and enzymatic digestion steps are absent from the standard PowerSoil protocol. These steps were incorporated into extraction method four (modified PowerSoil) by first homogenizing samples on the TissueLyser as in the phenol–chlorofom and Qiagen methods, and then incubating at 56°C overnight in 500 *μ*L PowerSoil bead solution, 60 *μ*L solution C1, and 100 *μ*g proteinase K. The digested samples were added to the PowerBead tubes and the extractions completed using the entire PowerSoil protocol. Again, all samples were eluted in 50 *μ*L of solution C6.

Total DNA concentrations of all samples were calculated on a Qubit fluorometer with the dsDNA High Sensitivity Assay Kit (Life Technologies Corp., Carlsbad, CA) using 5 *μ*L of extract. Samples below the detection limit (<0.20 ng/*μ*L) were assigned values of 0.20 ng/*μ*L for analysis and visualization.

### Insect samples

By comparing extraction protocols performed on different individuals, we introduced additional biological variation and reduced the sensitivity of our study to find minor differences in community structure due to biases in DNA extraction methodology. Subjecting the same sample to different extraction protocols would be more sensitive to detecting potential methodological influences, but, given that the influence of sample homogenization was part of what we aimed to test, this was not possible. However, due to their shared nests, close relatedness, and social food sharing through oral–oral trophallaxis, ants from the same colony are generally expected to host similar bacterial communities (Koch and Schmid-Hempel 2011; Koch et al. 2012, 2013; Hu et al. 2014). We utilized this intra-colony similarity for our study, assuming that bacterial communities would be similar enough within colonies to reveal influences of extraction methodology.

We used specimens from four species of ants: *Pseudomyrmex flavicornis*, *Pseudomyrmex nigrocinctus*, *Cephalotes varians*, and *Crematogaster rochai*. Ward (1993) and Longino (2003; http://academic.evergreen.edu/projects/ants/AntsofCostaRica.html) were used for ant species identification. All *Pseudomyrmex* and *Crematogaster* specimens were collected in June of 2012 at Santa Rosa Biological Station in the Área de Conservación Guanacaste in northwestern Costa Rica. *Cephalotes varians* specimens were collected from the Florida Keys between 2009 and 2011. Samples were stored in 95% ethanol until DNA extraction, which has been shown to be appropriate for preserving ant and ant-associated bacterial DNA (Moreau et al. 2013). All ants were surface sterilized in 5% bleach (0.25% weight/volume sodium hypochlorite solution) for one minute as in Sanders et al. (2014) and rinsed once with ddH_2_O before abdomens (metasomas) were removed from adult ants and extractions performed on just this part of the body. Larvae were extracted whole after surface sterilization.

For each of the extraction protocols, there were four types of ant material used: (1) a single adult abdomen, (2) three pooled adult abdomens, (3) a single larva, and (4) three pooled larvae. Larvae were not available for all colonies. All combinations of protocol and ant material were extracted from three colonies of each of the *Pseudomyrmex* species. DNA was extracted from adults only for a fourth colony of each *Pseudomyrmex* species, for four colonies of *Crematogaster rochai*, and for three colonies of *Cephalotes varians*. For one of the *Cephalotes varians* colonies, two extractions of each type were performed. In total, either eight or 16 extractions were performed for each colony. To serve as negative controls, three blank extractions with no insect material were also performed for each extraction protocol. Overall, 32 or 56 extractions were performed per ant species and 47 extractions were performed for each methodology, for a total of 188 DNA extractions. The same number and type of individuals from the same colonies were extracted using each protocol. A list of samples extracted per protocol is shown in Table 1. Note that individuals from particular colonies and life stages were paired across protocols for statistical analyses. All samples included are shown in Table S1.

**Table 1:** List of all extractions conducted for each extraction methodology. All sample characteristics were exactly replicated across extraction protocols

47 extractions per protocol
*Pseudomyrmex nigrocinctus*
3 colonies each with 4 sample types
1 individual adult
1 individual larva
1 pool of 3 adults
1 pool of 3 larvae
1 colony with 2 sample types
1 individual adult
1 individual larva
*Pseudomyrmex flavicornis*
3 colonies each with 4 sample types
1 individual adult
1 individual larva
1 pool of 3 adults
1 pool of 3 larvae
1 colony with 2 sample types
1 individual adult
1 individual larva
*Cephalotes varians*
2 colonies each with 2 sample types
1 individual adult
1 individual larva
1 colony with 4 sample types
2 individual adults
2 individual larvae
*Crematogaster rochai*
4 colonies with 2 sample types
1 individual adult
1 individual larva
3 blanks

### Bacterial quantification

We measured the amount of bacterial DNA present in extracts with quantitative PCRs (qPCRs) of the bacterial 16S rRNA gene. We used the 515f (5′-GTGCCAGCMGCCGCGGTAA) and 806r (5′ - GGACTACHVGGGTWTCTAAT) universal bacterial primers of the EMP to amplify the 16S rRNA gene from all bacteria and archaea present (http://www.earthmicrobiome.org/emp-standard-protocols/16s/). All qPCRs were performed on a CFX Connect Real-Time System (Bio-Rad, Hercules, CA) using SsoAdvanced 2X SYBR green supermix (Bio-Rad) and 2 *μ*L of DNA extract. Standard curves were created from serial dilutions of linearized plasmid containing inserts of the E. coli 16S rRNA gene. Melt curves were used to confirm the absence of qPCR primer dimers and we confirmed that amplification resulted from ant-associated communities and not contaminating DNA by comparing to no template controls. All samples were analyzed in triplicate and each standard dilution was included in triplicate in each qPCR reaction. The resulting triplicate quantities for each sample were averaged before calculating the number of bacterial 16S rRNA gene copies per microliter of DNA solution. These numbers were log_10_ transformed for all statistical analyses.

We used repeated measures analysis of variance (ANOVA) to assess the effect of extraction methodology on number of 16S rRNA gene copies. For this analysis, treatments were considered to be those samples from the same species, life stages, number of individuals, and colonies and samples from the same treatments were paired across extraction methodologies. Therefore, extraction methodology was the single independent variable and we measured the number of 16S rRNA gene copies in response to this variable. We also performed multifactor ANOVA to examine the effects of all other variables (species, colony, life stage, number of individuals) and their interactions. We confirmed our conclusions from these tests within each species using two-way ANOVAs with two independent variables, extraction method, and a sample-type variable that included life stage and number of individuals. Bonferroni-corrected pairwise *t*-tests were used to compare extraction kits, again pairing samples on the basis of all other variables. Differences in the number of bacterial 16S rRNA gene copies between life stages and number of individuals included in extractions were also determined by Bonferroni-corrected pairwise *t*-tests. The normality of the distribution of differences between all pairs of samples was confirmed prior to all statistical analyses.

### Bacterial community analysis

Bacterial 16S rRNA gene V4 sequencing was done on an Illumina HiSeq2000 (Illumina Inc., San Diego, CA) by the EMP following their standard sequencing protocols (Caporaso et al. 2012; http://www.earthmicrobiome.org/emp-standard-protocols/16s/) with the same primers used for qPCRs above with the addition of the necessary Illumina adapter sequences (515f: 5′ - AATGATACGGCGACCACCGAGATCTACACTATGGTAATTGTGTGCCAGCMGCCGCGGTAA, 806r: 5′ - CAAGCAGAAGACGGCATACGAGATAGTCAGTCAGCCGGACTACHVGGGTWTCTAAT; X's indicate barcode bases). We used forward reads for community comparisons, but the barcodes associated with the reverse primers were also sequenced so that samples could be demultiplexed. Samples for which sequencing yielded no or very few (<500) raw reads were considered sequencing failures.

QIIME (Caporaso et al. 2010b) was used for most analyses of the resulting data. Sequence data were simultaneously demultiplexed and quality filtered using default parameters. We called Operational Taxonomic Units (OTUs) with UCLUST open-reference 97% similarity clustering against the May 2013 release of the Greengenes database (DeSantis et al. 2006). OTUs represented by single sequences were excluded and representative sequences from the remaining non-reference OTUs were aligned against the Greengenes core set using PyNAST (Caporaso et al. 2010a). OTUs represented by sequences that failed to align frequently represented co-amplified ant 18S rRNA gene sequences and were also excluded from further analyses. The remaining sequences were then inserted into the Greengenes reference tree using ParsInsert (http://parsinsert.sourceforge.net/). Rarefaction curves were generated using mothur version 1.33.3 (Schloss et al. 2009). The OTU table was rarefied to a depth of 44,000 for further analyses. Alpha diversity was quantified using richness, Shannon diversity, and evenness as implemented in QIIME's equitability metric. Nonparametric *t*-tests with 1000 Monte Carlo permutations were used to compare alpha diversities between groups of samples. Comparisons of alpha diversities were based on averages of 1000 rarefactions. Random forests supervised learning classification as implemented in QIIME (Knights et al. 2011) as well as ANOSIM and PERMANOVA tests of Bray-Curtis, weighted UniFrac, and unweighted UniFrac beta diversity metrics were used to compare community level differences between treatments. Individual OTUs that appeared in at least 25% of samples were examined for relative abundance differences between treatments using ANOVA with Bonferroni correction. Community level differences were visualized using Principal Coordinates Analysis (PCoA) plots.

The proportions of samples that were successfully sequenced using each extraction methodology were compared with *χ*^2^ tests. We used separate *t*-tests to examine the relationship between sequencing success and both DNA concentration and bacterial 16S rRNA gene copies. The relationships between the number of sequence reads obtained from a sample and both the total DNA concentration and number of bacterial 16S rRNA gene copies were examined with Pearson's correlations.

## Results

### Bacterial quantification

**Figure 1:**
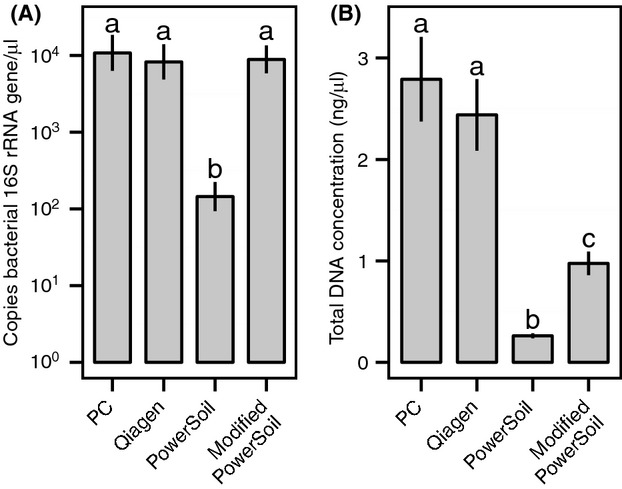
Measures of success for each bacterial extraction protocol (Phenol–chloroform: PC; Qiagen DNeasy Blood & Tissue Kit: Qiagen; PowerSoil DNA Isolation Kit: PowerSoil; and PowerSoil DNA Isolation Kit with the addition of a tissue homogenization and digestion step: modified PowerSoil). (A) Mean ± SE copies of bacterial 16S rRNA gene/*μ*L. (B) Mean ± SE total DNA concentrations (ng/*μ*L). Letters above bars show significant differences as determined by paired *t*-tests (*P* < 1 × 10^−4^).

**Figure 2:**
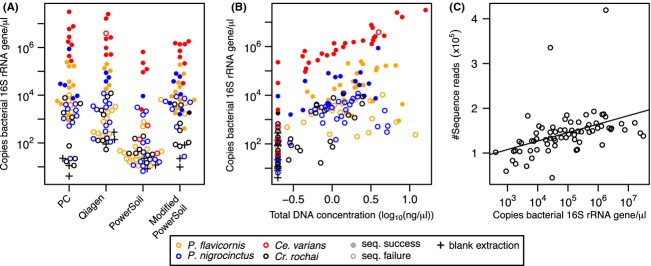
(A) Bacterial 16S rRNA gene concentrations (rRNA/*μ*L) recovered from all extracted samples by protocol (following notation as in Fig. 1). Colors correspond to ant species. Note the distribution of samples that we sequenced (filled circles) and that we failed to sequence (empty circles). (B) Total DNA concentration (log_10_(ng/*μ*L)) versus the concentration of the bacterial 16S rRNA gene (rRNA/*μ*L). (C) Correlation between concentration of the bacterial 16S rRNA gene (rRNA/*μ*L) and the number of reads (×10^5^) recovered for samples that we were able to sequence.

**Figure 3:**
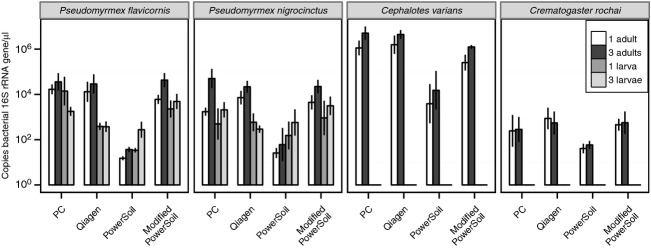
Mean ± SE concentration of the bacterial 16S rRNA gene (rRNA/*μ*L) by sample life stage and number of individuals for each species (following notation as in Fig. 1).

The effect of extraction methodology on the concentration of the bacterial 16S rRNA gene was highly significant (Repeated measures ANOVA, *F* = 61.5, *P* < 2 × 10^−16^; Fig. 1A). Multifactor ANOVA revealed that all other variables and several interactions were also significant (Table 2). Two-way ANOVAs on samples within each species confirmed that both extraction methodology and life stage were significantly associated with the number of 16S rRNA gene copies recovered in all species but *Crematogaster rochai* (Table S2). Qiagen, phenol–chloroform, and modified PowerSoil all had significantly larger numbers of 16S rRNA gene copies than the unmodified PowerSoil protocol (Bonferroni-corrected paired *t*-tests, *P* < 4.0 × 10^−10^; Figs. 1A, 2A, 3). Concordantly, sequencing success was significantly associated with extraction methodology (*χ*^2^-test, *χ *= 24.4, *P* = 2.0 × 10^−5^; Fig. 2A) and both the number of copies of the bacterial 16S rRNA gene found in each extraction (*t*-test, *t* = 14.04, *P* < 2.2 × 10^−16^; Fig. 2A) and the DNA concentration of the sample (*t*-test, *t* = 3.37, *P* = 0.001; Fig. 2B). In adult samples, pooling was an effective strategy for boosting 16S rRNA gene concentration as the extractions with three individuals had significantly greater quantities of bacterial DNA than single individual extractions (paired *t*-test, *t* = 4.6, *P* = 1.9 × 10^−5^; Fig. 3), but this was not the case in larvae (*t* = 1.45, *P* = 0.16; Fig. 3). In general, adult abdomens hosted significantly more bacterial DNA than whole larvae overall (paired *t*-test, *t* = 3.20, *P* = 0.003; Fig. 3), but not when using the unmodified PowerSoil extractions (Fig. 3). Total DNA concentration including both ant and microbe DNA was greatest in phenol–chloroform and Qiagen extractions and lowest in PowerSoil extractions (Bonferroni-corrected paired *t*-tests, *P* < 1 × 10^−4^; Fig. 1B). The number of copies of the bacterial 16S rRNA gene was significantly correlated with DNA concentration (Pearson's correlation, *t* = 6.70, *P* = 2.8 × 10^−10^; Fig. 2B), suggesting that effective extraction techniques are useful for all types of DNA present in these samples. The number of copies of the bacterial 16S rRNA gene was also correlated with the number of reads sequenced from each sample (Pearson's correlation, *t* = 3.88, *P* = 2.4 × 10^−4^; Fig. 2C). Different host species had significantly different ratios of bacterial 16S rRNA gene copy number to total DNA quantity (ANOVA, *F* = 59.21, *P* < 2 × 10^−16^), implying that overall bacterial load differs between these species (Fig. 3).

**Table 2:** Multifactor ANOVA on qPCR results including all interaction terms

Factor	df	*F*	*P* value
Kit	3	113.4	<2 × 10^−16^*
Species	3	198.0	<2 × 10^−16^*
Colony	11	10.8	2.4 × 10^−12^*
#Individuals	1	22.0	9.8 × 10^−6^*
Life stage	1	22.8	7.0 × 10^−6^*
Kit:Species	9	4.6	6.0 × 10^−5^*
Kit:Colony	33	2.4	0.0008*
Kit:#Individuals	3	1.0	0.39
Kit:Life stage	3	15.8	2.5 × 10^−8^*
Species:#Individuals	3	2.1	0.11
Species:Life stage	1	0.78	0.38
Colony:#Individuals	11	0.54	0.87
Colony:Life stage	4	4.7	0.002*

* Significant effect (*P* < 0.01).

### Bacterial community sequencing

We obtained a total of 11,842,139 sequence reads for our 188 samples and 10,246,746 of these passed quality filtering. However, 116 samples were assigned between zero and 331 sequences (Table S1). These samples were considered sequencing failures and were excluded from analyses. Between 44,830 and 419,430 (median = 144,625) sequences were assigned to the remaining 72 samples (Table S1). Rarefaction plots suggest that, while most of the taxa present in these samples were found at these sequencing depths, undiscovered diversity may remain (Fig. S1). Only a single *Crematogaster rochai* sample was successfully sequenced so this species was excluded from all bacterial community comparisons. Demultiplexed sequence reads have been submitted to NCBI's Sequence Read Archive under accession number SRP033241.

### Alpha diversity

Samples extracted using different methods were never significantly different in alpha diversity, the diversity within samples, whether species were analyzed together or individually (*t*-tests, *P* > 0.1). Results for all alpha diversity comparisons are shown in Table S3. Alpha diversity was significantly lower in both *Pseudomyrmex* species when compared to *Cephalotes varians* according to all three measures (richness, Shannon diversity, evenness; *t*-tests, *P* < 0.01), but alpha diversity did not differ significantly between *Pseudomyrmex* species. *P. flavicornis* was the only species for which both adult ant abdomens and whole larvae were successfully sequenced. Larvae had significantly higher alpha diversity within this species by all metrics (*t*-tests, *P* < 0.01). However, in both *Pseudomyrmex* species and *Cephalotes varians*, alpha diversity did not differ significantly between extractions of one and three individuals for adults or larvae (*t*-tests, *P* > 0.1), indicating that pooling at these scales is unlikely to artificially decrease recovered diversity, as has been previously reported (Manter et al. 2010). Alpha diversity was also significantly different between some colonies within *Cephalotes varians* and *P. flavicornis*. *Cephalotes varians* colony CSM1280 had a significantly lower alpha diversity than both CSM2194 and CSM1970 (*t*-tests, *P* < 0.01) by all metrics except richness. Conversely, *P. flavicornis* colony BER0512 had significantly higher richness than all other *P. flavicornis* colonies (*t*-test, *P* < 0.01), but other metrics were inconsistent. No pairs of *P. nigrocinctus* colonies significantly differed in alpha diversities.

At least three samples were successfully sequenced from each type of extraction methodology for *Cephalotes varians* colony CSM2194. Differences in alpha diversities between samples from this colony extracted with different methods were never significantly different (*t*-test, *P* > 0.1).

The presence of difficult to extract gram-positive bacteria did not seem to be heavily influenced by extraction protocol, though both Actinobacteria and Firmicutes were found in large numbers only rarely, and then usually within *Pseudomyrmex*. The maximum relative proportion of gram-positive bacteria from samples extracted with phenol–chloroform, Qiagen, PowerSoil, and modified PowerSoil methods was 80%, 20%, 1%, and 95%, respectively. The low maximum value reported for the standard PowerSoil extractions is likely due to the small number of these samples that were successfully sequenced. The maximum proportions of gram-positive bacteria for each extraction method limited to just those colonies with at least one successful PowerSoil extraction are more similar, at 0.06%, 0.5%, 1%, and 2%, respectively. Similarly, the much higher maximum proportions recovered from phenol–chloroform and modified PowerSoil extractions are both found in samples from the same *Pseudoymrmex* colony, BER0512, for which neither of the other two methodologies yielded any successful samples. Although we do not have extensive enough sampling to draw definitive conclusions on the gram-positive extracting abilities of each method, there do not appear to be drastic differences between them.

### Beta diversity

Supervised learning classification based on extraction methodology failed completely, suggesting that extraction methodology did not have a major impact on bacterial beta diversity, the species turnover between treatments. The ratio of baseline error rate that would occur if the classifier was completely random to the estimated error was 0.99, indicating that the classifier did no better than random. Classifying samples of each individual species by extraction methodology was similarly unsuccessful, with ratios of baseline error to estimated error of 1.0 or less; the classifiers did no better than random and often did worse. Supervised classification was also unsuccessful at distinguishing extraction methodology within *Cephalotes varians* colony CSM2194, where the baseline error to observed error ratio was 0.77, which is substantially worse than random. In contrast, genera (*Cephalotes* and *Pseudomyrmex*) were easily distinguished by supervised classification; only one out of 42 *Pseudomyrmex* and two out of 28 *Cephalotes* were classified incorrectly for a ratio of baseline error to observed error of 9.3. When attempting to classify the samples by species, all *Cephalotes* were classified correctly and only one *P. flavicornis* was misidentified as *Cephalotes varians*; but, while all *P. nigrocinctus* samples were successfully classified as a species of *Pseudomyrmex*, 11 of 16 were classified as *P. flavicornis*. Supervised classification effectively distinguished adult abdomens and larvae within *P. flavicornis* with an error ratio of 3.33, indicating that substantial differences exist between life stages. All abdomens were correctly classified as such but three of 10 larval samples were incorrectly identified as abdomens. Even colonies within species were distinguished by supervised classification. Only one sample from each of the three *Cephalotes varians* colonies was incorrectly assigned to colony, for an error ratio of 4.7. Colony assignment within the two *Pseudomyrmex* species was less successful, with error ratios of 1.0 for both analyses.

**Figure 4:**
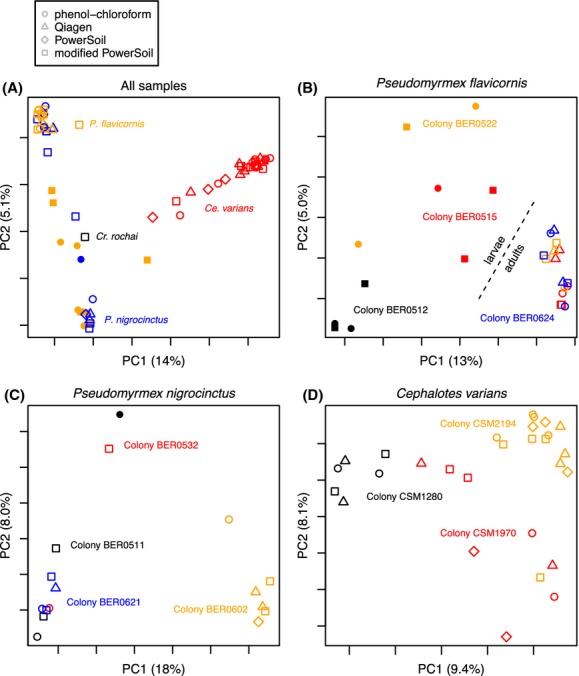
Principal coordinate plots of unweighted UniFrac distances between all sequenced samples (A), *Pseudomyrmex flavicornis* samples (B), *Pseudomyrmex nigrocinctus* samples (C), and *Cephalotes varians* samples (D). Colors correspond to different species in panel A and different colonies in B–D. Adults and larvae are represented as open and filled symbols, respectively.

No pattern was apparent between extraction protocols in PCoA plots using unweighted UniFrac distances between samples (Fig. 4), but differences between species, colonies, and life stages were clear. Neither ANOSIM nor PERMANOVA analyses found significant differences between extraction methodologies when all samples were included (*P* > 0.1; Fig. 4), regardless of the beta diversity metric used. The lack of significant effect held true when only samples within individual species were compared (*P* > 0.1; Fig. 4). However, Species hosted significantly different communities according to all beta diversity metrics (ANOSIM, PERMANOVA, *P* = 0.001; Fig. 4). Within species, colonies hosted significantly different communities (*P* < 0.005; Fig. 4). While extractions from three abdomens and one abdomen were not significantly different for *P. nigrocinctus* or *Cephalotes varians* (*P* > 0.1), there were significant differences between the larval and adult extractions within *P. flavicornis* (*P* = 0.001; Fig. 4). The results from all statistical tests of beta diversity differences can be found in Table S4.

### Core community comparison

Of 199 OTUs that occurred in at least 25% of samples, none were significantly different in abundance between samples extracted with different extraction protocols (ANOVA, Bonferroni-corrected *P* > 0.3). Within *Cephalotes varians* samples, 700 OTUs were compared; again, none were significantly different in abundance between samples extracted with different methodologies (*P* > 0.1). Similarly, none of the 199 OTUs compared in *P. nigrocinctus* were significantly different between extraction treatments. Of the 164 OTUs compared between extraction methodologies in *P. flavicornis* samples, two were significantly different in abundance (*P* < 0.05). Both of these taxa were classified as Alphaproteobacteria and had 10-fold greater relative abundance in Qiagen extraction samples than other samples. No taxa differed significantly in abundance between extraction protocols in *Cephalotes varians* colony CSM2194. Differences between species were also apparent when testing individual OTUs. Of 199 tested OTUs, 154 were significantly different in abundance between the genera *Cephalotes* and *Pseudomyrmex* (*P* < 0.01). Within *Cephalotes*, 700 OTUs were examined and 26 OTUs were different in abundance between colonies (*P* < 0.01). In *P. flavicornis*, only two OTUs were significantly different between colonies and in *P. nigrocinctus*, six were significantly different (*P* < 0.01). Within *P. flavicornis*, six OTUs differed significantly in abundance between larval and adult extractions (*P* < 0.01).

## Discussion

The importance of standardized DNA extractions for microbial community characterization is clear (de Lipthay et al. 2004; Carrigg et al. 2007; Feinstein et al. 2009; Willner et al. 2012); however, contrary to popular opinion among researchers, our study suggests that for ants, the DNA extraction protocol often does little to change the recovered community structure when examining 16S rRNA amplicon data. Rather, these differences in protocols have a much greater impact on whether bacterial metacommunities can be sequenced at all. As has been reported previously for differences in sample storage condition (Lauber et al. 2010; Rubin et al. 2013), we find that extraction protocol has little influence on bacterial community composition relative to sample source. In this study, bacterial community structure and composition in ants of the same species and colony were most similar to each other, regardless of DNA extraction methodology. However, some of the DNA extraction protocols led to a significant increase in failed 16S rRNA amplicon sequencing runs, effectively preventing the characterization of certain communities by sequencing. Combined with the lack of strong biases introduced by extraction protocols, these findings suggest that minor methodological optimizations can justifiably take precedence over universal standardization.

Recently, the MO BIO PowerSoil kit has gained traction as the standard technique for extracting bacterial DNA from environmental samples, including for two of the largest microbiome initiatives, the EMP and the Human Microbiome Project. This protocol was originally developed to extract community DNA from soil samples, but has been successfully deployed in a number of non-soil studies (e.g., Costello et al. 2009; Suen et al. 2010; Wu et al. 2011). Unfortunately, the standard protocol is clearly suboptimal for extracting DNA from intact insects. Although the PowerSoil kit includes a lengthy vortexing step with ceramic beads, the apparent violence of such vigorous mixing is insufficient to disrupt the exoskeleton and extract the majority of the bacteria in ants. In fact, we visually identified both intact larvae and whole abdomens in some samples after this vortexing step (data not shown). Even soft-bodied larvae yield far less bacterial DNA when extracted with this protocol, though the difference between methods was certainly less pronounced for larvae than for adults, suggesting that the rigid cuticle of the adults is largely responsible for the decreased amount of extracted bacterial DNA. These extractions yielded orders of magnitude fewer copies of the bacterial 16S rRNA gene than other methods and, consequently, had significantly lower sequencing success. The relatively simple additional steps of tissue pulverization and digestion radically improved the extraction results, and rescued a large number of samples from a bacterial DNA concentration too low to sequence without risking the fidelity of the resulting community profiles.

Despite the clear drawbacks of the standard PowerSoil protocol, the modified technique yielded the largest number of successfully sequenced extractions. Our results indicate that the concentration necessary for dependable sequencing is around 10,000 copies of target DNA per microliter, yet a substantial fraction of the modified PowerSoil samples with bacterial 16S rRNA gene counts below this cutoff were successful. This increased frequency of success may be due to the removal of inhibitors that other techniques fail to eliminate. The removal of PCR inhibitors is likely to be particularly important for the characterization of bacterial communities from certain types of samples, such as insects that feed on large volumes of plant-derived resources (e.g., termites, dung beetles).

Some insect-associated bacterial communities are too depauperate to be characterized using any of the techniques explored here. While sequencing was successful for nearly all samples of *Cephalotes varians*, which is known to host a large population of gut bacteria (Russell et al. 2009; Kautz et al. 2013a; Hu et al. 2014; Sanders et al. 2014), we recovered sequences from just one of 32 extractions of *Crematogaster rochai*. *Crematogaster* had a bacterial 16S rRNA gene copy number to total DNA ratio that was on average 100-fold lower than *Cephalotes*, suggesting a much lower density of bacteria in the gut. Differences in ant gut morphology or the predominance of fundamentally difficult to extract bacteria (e.g., with particularly resilient cell walls) in *Crematogaster* could also contribute to these differences. Regardless of cause, this disparity is likely the underlying reason that bacterial 16S rRNA gene copy number estimates were much more successful at predicting sequencing success than total DNA concentration. Ideally, the concentration of bacterial DNA could be used both for predicting sequencing success and to standardize across samples, increasing the evenness of sequencing coverage. This approach has the substantial additional benefit of revealing biologically relevant but understudied patterns in the absolute abundance of host-associated microbes (Engel and Moran 2013). For insects with low bacterial titers, such as *Crematogaster rochai*, large pools of individuals may be required to describe those microbes that are present.

The guts of insects and other hard-bodied arthropods are physically very different than the feces often examined in studies of larger animals. Short of time-consuming and difficult dissections, which have been successfully done by some researchers (Martinson et al. 2012; Kautz et al. 2013b; Hu et al. 2014), insect guts are almost always surrounded by a rigid exoskeleton. Extraction protocols do not always include a step capable of disrupting these exoskeletons and the tissues within, yet this is essential if we hope to sequence the DNA of the endosymbiotic bacteria present. Environment-specific modifications to extraction protocols will likely be necessary for examining many types of microbiomes, not just insect associates. While these modifications should always be minimized, some flexibility will encourage researchers from a wide range of fields to explore bacterial metacommunities, drastically increasing the number of environments studied and expanding our understanding of Earth's microbiome.

## Supporting Information

Figure S1 Rarefaction curves for every successfully sequenced sample colored by species.

Table S1 Characteristics, DNA concentrations, qPCR results, and sequence read numbers of all extracted samples.

Table S2 Results of two-way ANOVAs comparing 16S rRNA copy numbers between methodologies and sample types within each species.

Table S3 Results of *t*-tests comparing alpha diversity between all groups of samples.

Table S4 ANOSIM and PERMANOVA comparisons of beta diversity between treatments in all subsets of samples.
