# Supplementary figures and images for: DNA extraction protocols cause differences in 16S rRNA amplicon sequencing efficiency but not in community profile composition or structure
Source: Microbiologyopen. 2014 Sep 26;3(6):910–21. doi: 10.1002/mbo3.216 (PMC4263514; doi:10.1002/mbo3.216)

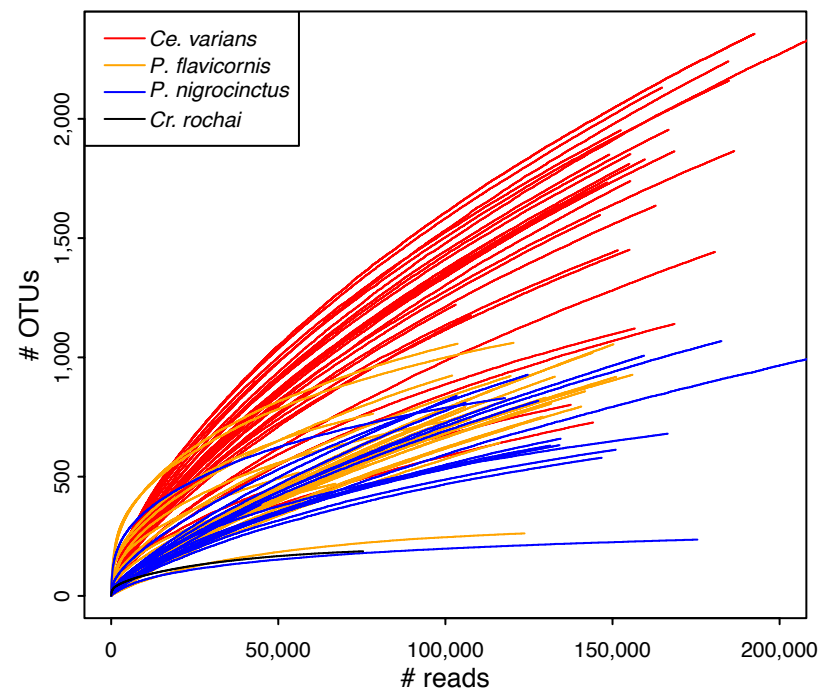

Supplement: Figure S1 — Rarefaction curves for every successfully sequenced sample colored by species. [file mbo30003-0910-sd1.pdf]
